# A modified banding technique: experience of a center

**DOI:** 10.1590/2175-8239-JBN-2020-0046

**Published:** 2020-11-11

**Authors:** Sofia S. G. Cerqueira, Joana M. Ferreira, Mónica R. Fructuoso, Catarina Eusebio, Rui A. Castro, Teresa M. Morgado

**Affiliations:** 1 Centro Hospitalar Trás-os-Montes e Alto Douro, Hospital de Vila Real, Serviço de Nefrologia, Vila Real, Portugal.; 2 Hospital da Senhora da Oliveira, Serviço de Angiologia e Cirurgia Vascular, Guimarães, Portugal.

**Keywords:** Renal Dialysis, Arteriovenous Fistula, Athletic Tape, Diálise Renal, Fístula Arteriovenosa, Fita Atlética

## Abstract

**Background:**

A well-functioning vascular access is vital to patients on regular hemodialysis. Banding the access is indicated in high-flow-associated steal syndrome. It allows for the reduction of access flow while maintaining distal limb perfusion. Nonetheless, this procedure has some limitations as it can cause hemorrhage, infection, aneurysm formation, thrombosis of access in cases of *overbanding*, or otherwise insufficient reduction of vascular flow. Other surgical techniques to achieve the same benefit would be useful.

**Methods:**

We performed a modified banding technique without endovascular placement of the angioplasty balloon, which is a viable alternative to other techniques. This surgery was performed in patients on chronic dialysis with steal syndrome. Pre- and post-operative access flows were measured and resolution of symptoms was recorded. Primary patency rate was defined as the intervention-free access survival from the operative time.

**Results:**

We verified that this technique allowed for access flow reduction in all our six patients, with total resolution of symptoms in all patients. Primary patency rate at 12 months was 100%. No major complications were noted during our follow-up.

**Conclusions:**

This technique allows for correction of high-flow arteriovenous fistulas in an efficient and safe way, and can be a viable alternative to other banding procedures.

## RESUMO

**Introdução:**

Um acesso vascular em bom funcionamento é vital para pacientes em hemodiálise regular. A bandagem do acesso é indicada na síndrome de roubo associada a alto fluxo. Permite a redução do fluxo de acesso enquanto mantém a perfusão distal do membro. No entanto, este procedimento tem algumas limitações, pois pode causar hemorragia, infecção, formação de aneurisma, trombose de acesso em casos de excesso de bandagem (*overbanding*) ou, de outra forma, redução insuficiente do fluxo vascular. Outras técnicas cirúrgicas para obter o mesmo benefício seriam úteis.

**Métodos:**

Foi realizada uma técnica de bandagem modificada sem colocação endovascular do balão de angioplastia, que é uma alternativa viável às outras técnicas. Esta cirurgia foi realizada em pacientes em diálise crônica com síndrome de roubo. Os fluxos de acesso pré e pós-operatório foram medidos e a resolução dos sintomas foi registrada. A taxa de permeabilidade primária foi definida como a sobrevivência do acesso livre de intervenção desde o tempo operatório.

**Resultados:**

Verificamos que essa técnica permitiu redução do fluxo de acesso em todos os nossos seis pacientes, com resolução total dos sintomas em todos os pacientes. A taxa de patência primária em 12 meses foi de 100%. Nenhuma complicação maior foi observada durante nosso acompanhamento.

**Conclusões:**

Esta técnica permite a correção de fístulas arteriovenosas de alto fluxo de forma eficiente e segura, podendo ser uma alternativa viável a outros procedimentos de bandagem.

## INTRODUCTION

The Vascular Access Society has defined a high-flow access as one with a flow between 1-1.5 L/minute or a Qa that is >20% of the cardiac output.

High-flow accesses greatly affect the systemic hemodynamics of patients performing regular hemodialysis^1^. The characteristic hyperdynamic circulation in these cases leads to increased cardiac output and decreased peripheral vascular resistance. Clinical presentation varies from acute decompensation of chronic heart failure to symptoms of low hand perfusion pressure, although the prevalence of each one of the complications is still unknown^2^. In severe cases, hemodialysis access-induced distal ischemia (HAIDI, otherwise known as "steal syndrome") can develop. These symptoms are more frequently associated with proximal (brachial-based) fistulas as opposed to distal (radiocephalic) ones, and if not adequately treated can carry risk of limb loss^3^. Risk is also higher in women and patients with diabetes. Generalized arteriosclerotic disease, carpal tunnel syndrome, and secondary hyperparathyroidism seem to associate with worsened steal symptomatology.

Banding the access is indicated in high-flow-associated steal syndrome. This technique creates a high resistance area within the arteriovenous access, producing a "functional stenosis". It allows for the reduction of access flow while maintaining distal limb perfusion. The RUDI (revision using distal inflow) and the MILLER (4) (minimally invasive limited ligation endoluminal-assisted revision) procedures are modifications of the original banding techniques. The MILLER procedure can be performed in cases of high-flow vascular accesses. It consists of exposing the access vein or graft near its anastomosis to the artery, inserting and inflating an angioplasty balloon within the vessel and tying a non-resorbable suture around the access to redefine the vessel diameter to a smaller size. Main complications of this technique are: hemorrhage, infection, aneurysm formation, thrombosis of access in cases of *overbanding,* or otherwise insufficient reduction of vascular flow. 

The authors describe a modified banding procedure that represents a viable alternative to other banding procedures. The technique was performed at our center in 6 patients with high-flow arteriovenous fistulas and distal ischemia, often with cardiac overload.

## METHODS

### PATIENT SELECTION

A retrospective analysis of 6 modified MILLER surgeries performed in six patients from our hospital's chronic dialysis program (Hospital de Vila Real, in Vila Real, Portugal) between 2016 and 2018 was carried out. Our hospital's dialysis program comprised 60 patients at that time. Selected patients had symptomatic "steal syndrome" and high-flow accesses.

We performed a retrospective analysis of success rates, procedure complications, and post-operative patency. All the patients had pre-operative flow measurements with Doppler ultrasound (using Philips CX50 Ultrasound) as well as measurement of vessel diameter to determine size of the angioplasty balloon to be used. Our aim was to reduce vessel size to 50% of its previous size. Access flow was also measured 6 months post-surgery to evaluate flow reduction.

### OUTCOME AND DEFINITIONS

Pre-operative access flow was measured immediately before surgery. Post-operative access flows were measured 6 months after surgery. A successful procedure was defined as one bearing resolution of symptoms with preserved arteriovenous thrill. Resolution of symptoms meant absent dyspnea, dyspnea under exercise, or pain in the hand or forearm during dialysis or at rest. Primary patency rate was defined as the intervention-free access survival from the operative time.

### TECHNIQUE DEFINITION

**Figure 1:**
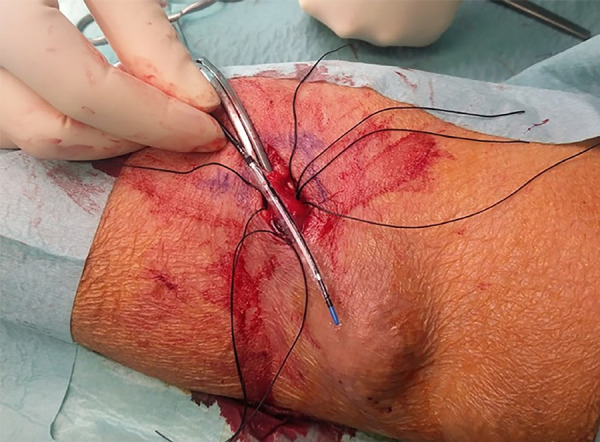
Clamped cephalic vein. Angioplasty balloon lateral to the vein.

**Figure 2:**
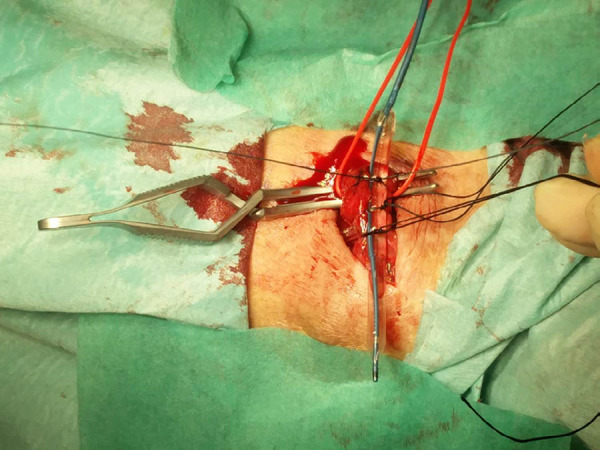
Angioplasty balloon lateral to the vein. Sutures in position.

**Figure 3:**
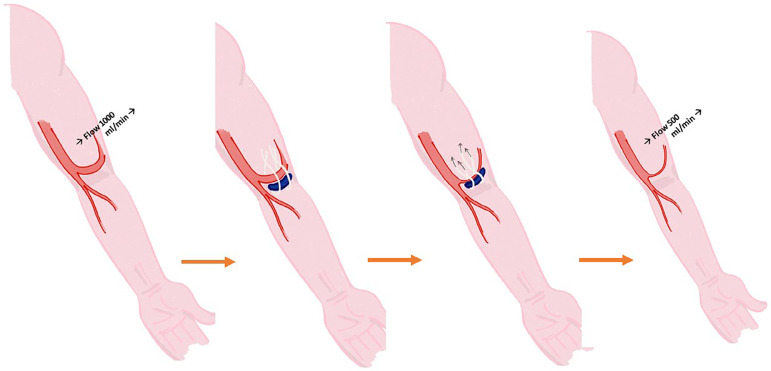
Flow is reduced without intravascular placement of an angioplasty balloon.

A surgical incision was performed under local anesthesia in the venous juxta-anastomotic segment. The anastomosis and 3 to 4 cm of the vein were exposed. The arterialized vein was clamped after the arteriovenous anastomosis to interrupt flow and the inflated angioplasty balloon was then placed in an extraluminal position next to the collapsed vein at the banding site (Figure 1). Three 2-0 polyfilament sutures were placed under the vein, at a distance of 0.5 cm each of one another. These sutures were used to hold tight the expanded balloon and the collapsed vein (Figure 2). The balloon was then deflated and removed, the vein was declamped, and after flow restoration the vein acquired the balloon size at the banding site (Figure 3). The fistula's thrill and distal perfusion of the hand were monitored intra-operatively using clinical inspection and palpation.

### STATISTICAL ANALYSIS

Differences between pre- and post-operative groups were determined using the paired samples t-test. A p < 0.05 was considered significant. SPSS 16.0 software (SPSS Inc, Chicago, Ill) was used for statistical analysis.

## RESULTS

Average age was 61 ± 13 years, with most of our population being of female gender (67%), as expected. Half of these patients were diabetic (as shown on Table 1). All of our patients had initial access flow superior to 1500 mL/min and diagnostic criteria for "steal syndrome". Two thirds of arteriovenous fistulas were brachio-cephalic and the remaining were brachio-basilic fistulas. There was no arteriovenous graft. Mean pre-operative vein diameter was 9 ± 3 mm and the angioplasty balloons used were 4, 5, or 6 mm in diameter. Mean follow up was 19 months.

**Table 1:** Demographic data

Patient number	Gender	Age	Cardiovascular disease	Diabetes	Pre-op Kt/V
**#1**	Male	47	No	No	1.46
**#2**	Female	80	Yes	No	1.8
**#3**	Female	49	Yes	Yes	NA
**#4**	Female	75	No	Yes	1.5
**#5**	Female	64	No	Yes	1.9
**#6**	Male	61	Yes	No	1.29

Abbreviations: NA, not available; Pre-op, pre-operative

Success rate was 100%. There was a reduction of mean pre-operative flow of 2163 +- 509 ml/min to 1495 +- 222 ml/min 6 months after surgery. This difference was statistically significant (p<0.05). All patients had immediate post-operative symptom relief (Table 2). Rate of complications was 33% (n=2): one infection and one aneurysm formation. Primary patency at 12 months was 100%.

**Table 2:** Pre-operative and post-operative data

Patient number	Type of access	Pre-operative flow	Post-operative flow	Resolution of symptoms
**#1**	Brachio-cephalic fistula	2500	1800	Yes
**#2**	Brachio-cephalic fistula	2100	1700	Yes
**#3**	Brachio-cephalic fistula	1900	1200	Yes
**#4**	Brachio-basilic fistula	3000	1500	Yes
**#5**	Brachio-basilic fistula	1900	1370	Yes
**#6**	Brachio-cephalic fistula	1580	1400	Yes

## DISCUSSION AND CONCLUSION

Patients on regular hemodialysis depend on a well-functioning vascular access. However, due to increasingly prevalent atherosclerosis, these accesses are difficult to create, and their viability is frequently threatened by findings such as stenosis or dilations at the arteriovenous anastomosis. High-flow accesses due to such arteriovenous dilations can lead to symptoms like HAIDI, which can be very debilitating.

Clinical presentation varies, and there is no direct correlation between access flow and clinical manifestations. Patients with high-flow dialysis accesses can present with acute decompensation of chronic heart failure, pulmonary hypertension, central vein stenosis, or symptoms of low hand perfusion. Older age, prevalence of atherosclerosis, and comorbidities such as diabetes in chronic kidney patients on hemodialysis prompts us to find new ways to deal with more frequent cases of HAIDI.

The main goals of the steal syndrome correction are to preserve the access while simultaneously improving peripheral arterial circulation. In order to do so, one has to achieve an equipoise between reduction of intra-access flow while maintaining sufficient distal perfusion of the hand.

Since the 1970s, banding the access has been the preferred treatment in these cases. Since then, new surgical techniques have been developed 4 ^,^ 5 ^,^ 6, aiming to achieve the same benefits, such as the Distal Revascularization with Interval Ligation (DRIL), creation of a more proximal arteriovenous anastomosis (PAVA), or the Revision Using Distal Inflow (RUDI). Minimally Invasive limited ligation endoluminal-assisted revision (MILLER) is a more recent endovascular procedure that uses an angioplasty balloon of a selected size to create a "functional stenosis" within the anastomosis and gain a defined reduction in the vessel diameter.

However, all of these techniques have some drawbacks, and success rates are variable, as are access patency at 12 months (Odland et al^7^ reported a 100% relief of symptoms but a 38% access patency at 12 months in 16 patients, while Meyer et al^8^, in 7 patients, reported 100% symptom relief and access patency at 12 months). Bermann et al^9^, Knox et al^10^, and Lazarides et al^11^ reported their experience with DRIL surgery that achieved variable degrees of symptom relief and 1-year access patency rates that ranged from 83 to 94 or 100%. Leake et al^12^ reported their center's experience and outcomes on 201 patients with "dialysis-associated steal syndrome" using several techniques. Symptom relief ranged from 75% (using banding) to 98% (using DRIL) (P = 0.005). Fistula preservation was 0% for ligation, 100% for DRIL, 95% for RUDI, and 89% for banding (P < 0.01). Complications were highest in the banding (49%) and RUDI (37%) groups. According to this, they state that DRIL should be considered the preferred procedure for management of these situations, but to do so patients should be able to tolerate a major operation.

New techniques are needed to achieve the best effectiveness with the lowest complication rates. Schneider et al^13^, in 2006, reported their experience on 25 patients using a banding procedure that uses a T-band conformation patch to reduce access flow. However, fistula thrombosis occurred in two patients, and primary patency rate reported at 1-3 months was 90%.

Modified banding techniques such as the MILLER procedure have proved effective in treating these cases^14^^,^ 15 ^,^ 16 but are not widely available. They are also more invasive than our presently reported technique and more dependent on operative skills and experience. The main advantages of our technique was the lesser invasiveness, easiness of procedure, and substantially smaller risk of thrombosis, while upholding equivalent efficacy.

In our limited experience, this modified banding technique demonstrated to be efficient and safe, showed an elevated success rate, a high long-term patency, and was a viable option in cases of high-flow-associated steal syndrome.
